# Quatsomes as versatile fluorescent nanocarriers: stable Eosin Y loading and FRET with a membrane dye

**DOI:** 10.1039/d6na00124f

**Published:** 2026-05-15

**Authors:** Andrea Delledonne, Guillem Vargas-Nadal, Giacomo Cotelli, Nora Ventosa, Mariana Köber, Cristina Sissa

**Affiliations:** a Dipartimento di Scienze Chimiche, Della Vita e Della Sostenibilità Ambientale, Università di Parma Parco Area Delle Scienze 17A 43124 Parma Italy cristina.sissa@unipr.it; b Institute of Materials Science of Barcelona (ICMAB-CSIC), Universitat Autonoma de Barcelona 08193 Barcelona Spain mkober@icmab.es; c Centro de Investigación Biomédica en Red in the Subject Area of Bioengineering, Biomaterials and Nanomedicine (CIBER-BBN) 28029 Madrid Spain

## Abstract

Fluorescent nanoprobes are key components in advanced bioimaging and optical sensing, enabling enhanced brightness, photostability, and multifunctionality beyond what is achievable with molecular dyes. Using multiple dyes in one nanocarrier boosts signal and enables multi-color imaging in a single system. However, the development of nanoplatforms capable of stably incorporating multiple fluorescent probes with different solubility and physicochemical properties remains a significant challenge. In this work, we demonstrate the efficient co-encapsulation of a hydrophilic dye (Eosin Y) and a hydrophobic dye (DiD) in quatsomes (QSs), a class of stable, non-liposomal nanovesicles composed of ionic surfactants and sterols. QSs are multifunctional nanocarriers of interest for drug delivery and bioimaging, since they can encapsulate molecules with different functionalities. Two different strategies are proposed for the loading of Eosin Y: (a) pre-assembly loading during the preparation of nanovescicles, and (b) post-assembly loading (incubation) of nanovescicles with a solution of the dye. While the hydrophobic probe DiD easily inserts into QS membranes thanks to its long alkyl chains, our results show that a hydrophilic dye like Eosin Y can also be efficiently and stably incorporated, even after vesicle formation. This opens the possibility of tuning the emission properties of QSs on demand, provided that suitable hydrophilic dyes with appropriate structural affinity for the QS system are employed. Moreover, Eosin Y and DiD are dyes compatible for Förster resonance energy transfer (FRET). FRET between two fluorophores on the same carrier provides a sensitive readout of nanoscale interactions, converting molecular proximity into a measurable optical signal. Together, these results position QS as a versatile and modular nanoplatform for multicolor bioimaging and optical sensing, combining stability with on-demand tunability of their emission properties.

## Abstract 2

Quatsomes, non-liposomal nanovesicles made of ionic surfactants and sterols, co-encapsulate hydrophilic Eosin Y and hydrophobic DiD by pre- or post-assembly loading, enabling tunable emission and FRET optical readouts.

## Graphical abstract

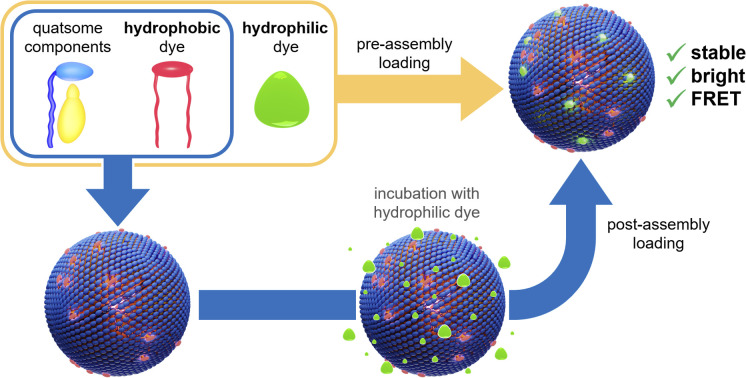

## 1 Introduction

Fluorescent probes play a central role in bioimaging, chemical sensing, and optoelectronics. Despite decades of development, the field remains highly active because emerging applications impose requirements that extend beyond efficient light emission. In particular, next-generation probes must combine high brightness and photostability with chemical stability in complex environments, while also reporting on local structure and interactions at the nanoscale.^1,2^ In this context, ideal fluorescent probes should exhibit tunable emission properties, environmental sensitivity, and the possibility to integrate multiple functionalities within a single system. Nanostructured fluorescent platforms provide an effective strategy to meet these requirements, enabling enhanced optical performance and multifunctionality that are difficult to achieve with molecular dyes alone.^3,4^ Quatsomes (QSs) are thermodynamically stable non-liposomal nanovesicles composed of ionic surfactants, typically quaternary ammonium surfactants, and sterol derivatives such as cholesterol.^5,6^ These small unilamellar nanovesicles (SUVs), with diameters typically below 200 nm, maintain structural integrity even under varying conditions, such as fluctuations in pH, temperature, and ionic strength.^7–9^ Their stability and uniformity make them ideal candidates for a variety of applications, particularly in drug delivery and bioimaging, with promising results in preliminary biocompatibility assessments.^10^ To prepare QSs, several techniques are employed, including thin-film hydration, reverse-phase evaporation, and ethanol injection. However, challenges such as the formation of sterol-rich domains can hinder self-assembly, and post-formation steps like sonication^11^ and extrusion^12^ are often necessary to control size distribution and homogeneity. A notable advancement in this area is the DELOS-SUSP process,^13^ which usess compressed CO_2_ for the robust and scalable preparation of QSs.^14^ Among the various QSs formulations, those based on the cationic surfactant cetyltrimethylammonium bromide (CTAB) and cholesterol are the most extensively studied, owing to their structural robustness, ease of preparation, and well-understood self-assembly.^5,14,15^ These nanovesicles feature positively charged inner and outer surfaces, a hydrophobic membrane bilayer, and an aqueous interior, known as the lumen, enabling the encapsulation of both hydrophilic and hydrophobic molecules.

CTAB-QSs have demonstrated high compatibility with various functional molecules, including hydrophobic fluorophores such as diketopyrrolopyrroles,^16^ fluorene derivatives,^8^ and cyanines,^9^ as well as larger nanomaterials such as silicon nanocrystals^17^ and proteins.^18^ Notably, CTAB-QSs have proven highly effective for Förster Resonance Energy Transfer (FRET) studies when loaded with DiI (1,1-dioctadecyl-3,3,3,3-tetramethylindocarbocyanine perchlorate) and DiD (1,1′-dioctadecyl-3,3,3′,3′-tetramethylindodicarbocyanine perchlorate), a pair of membrane-anchored lipophilic cyanine dyes.^19,20^ FRET is a non-radiative energy transfer mechanism occurring between an energy donor and an energy acceptor fluorophore, typically separated by 1–10 nm, and its efficiency is sensitive to both inter-dye distance and relative orientation. This makes it a powerful tool for probing nanoscale organization and dynamics in soft matter systems.^21,22^

Despite this progress, strategies for incorporating hydrophilic dyes into CTAB-based quatsomes have remained largely unexplored. The first reported system involves fluorescein,^23^ which failed to establish a stable interaction with the nanoparticles, highlighting the need for alternative approaches to achieve effective loading of hydrophilic cargoes. A successful example has only just emerged, involving the incorporation of indocyanine green, a water-soluble dye.^24^ Still, the full potential of QSs as multifunctional nanoprobes has not been fully exploited, particularly with respect to the simultaneous incorporation of multiple dyes with different solubility and physicochemical properties.

**Fig. 1:**
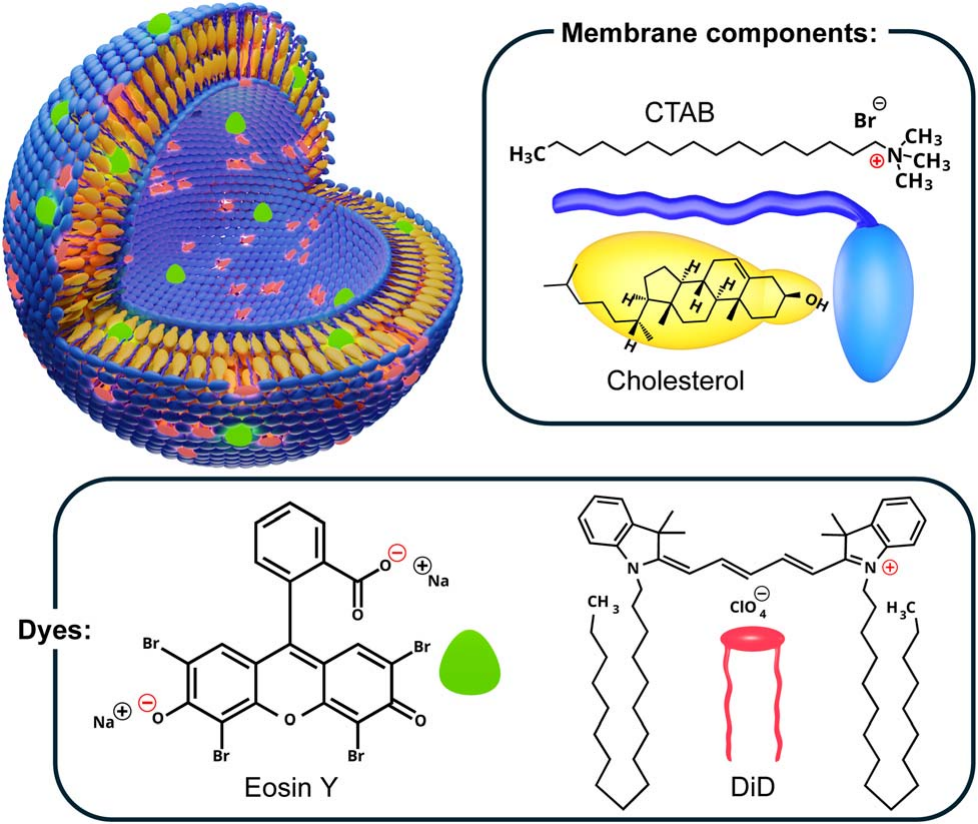
Graphic representation of the dye-loaded CTAB-QSs studied in this work, showing the molecular structures of their membrane components (CTAB and cholesterol) and the two dyes, DiD and Eosin Y (EoY).

In this work, we compare two strategies for incorporating Eosin Y (EoY), a water-soluble xanthene dye, into CTAB-QSs: pre-assembly loading during nanoparticle formation *via* the DELOS-SUSP method, and post-assembly incubation of nanovesicles. The resulting systems are referred to as pre-assembly and post-assembly loaded (or incubated) QSs, respectively. EoY was selected as a model hydrophilic probe due to its excellent water solubility and valuable spectroscopic properties.^25^ EoY has a long-standing track record in diverse research areas, including bioimaging,^26^ photodynamic therapy,^27^ and analytical chemistry^28^ thanks to its intense absorption and fluorescence in the green spectral region and its sensitivity to local microenvironments.^29^ The chemical structures of EoY and the membrane components used for vesicle formation are shown in Fig. 1. Here, we provide a detailed analysis of the prepared nanovesicles through cryogenic transmission electron microscopy (cryo-TEM), dynamic and electrophoretic light scattering (DLS and ELS), and absorption and fluorescence spectroscopy. Finally, we assess the FRET performance of hybrid nanovesicles simultaneously loaded with two dyes: EoY, the energy donor, and DiD, a hydrophobic, red-emitting fluorophore known to integrate into the lipid membrane^9^ and suitable as energy acceptor for EoY. This dual loading strategy demonstrates the ability of CTAB-QSs to co-encapsulate both hydrophilic and hydrophobic cargos, enabling intravesicular FRET processes and expanding their utility as multifunctional optical nanoplatforms.

## 2 Results and discussion

Colloidal formulations of CTAB-QS were prepared following both the pre- and post-assembly loading methods in order to encapsulate the hydrophilic dye EoY. Samples were prepared both in the presence and in the absence of the hydrophobic dye DiD. The encapsulation of DiD in QS membranes was extensively investigated by some of us: it is well established that long alkyl chains of the DiD facilitate its incorporation into the lipid membrane.^9,19,20^ Moreover, DiD and EoY are compatible for FRET, thanks to the overlap of the emission spectrum of EoY with the absorbance spectrum of DiD, resulting EoY the energy donor and DiD the energy acceptor (Fig. S1, SI).

A detailed overview of the concentrations of the dyes and membrane components in each prepared sample is reported in the SI (Table S1, SI), while experimental details on the two preparation methods can be found in Section 4.1.

**Fig. 2:**
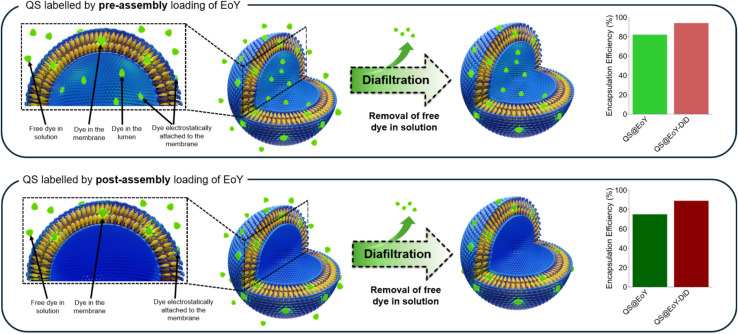
Scheme of the diafiltration purification process, which removes free dye in solution, along with ethanol and any residual surfactant or cholesterol not incorporated into the membrane of the nanovesicles. The possible locations of the hydrophilic EoY in the QSs dispersion prepared *via* pre-assembly loading (top panel) and post-assembly loading (bottom panel) are also highlighted, together with the corresponding E.E. after diafiltration.

Following preparation, all samples were purified by diafiltration (Fig. 2), which effectively removes free dye molecules from the colloidal dispersion. The purified samples were then characterized using DLS, ELS, cryo-TEM, as well as UV—visible and fluorescence spectroscopy. The encapsulation efficiency (E.E.) of both EoY and DiD was evaluated by quantifying the dye retained in the QS dispersions after diafiltration (as described in Section 4.2.1), and the resulting E.E. values are summarized in Fig. 2a and Table S1, SI.

Notably, EoY is not removed significantly from the QSs after diafiltration. Regardless of the preparation method, both DiD and EoY exhibited E.E. values in the range of 70–90%, which is comparable to previous results reported for CTAB/cholesterol QSs loaded with DiI and DiD.^9,19^

Due to its physicochemical properties, EoY can localize in different regions within the QSs (Fig. 2): it may be solubilized in the aqueous lumen, electrostatically bound to the membrane surface, or embedded within the bilayer. While EoY is water-soluble and could initially exist as free dye in solution, diafiltration effectively removes unbound molecules as long as no dissociation equilibrium is present. Additionally, since QSs are already assembled before exposure to EoY in the post-assembly method, the lumen should not be accessible during this procedure (except in case of slow diffusion across the bilayer membrane).

The acid-base properties of EoY further elucidate its interaction with the QS membrane. With p*K*_a_ values of 2.0 and 3.8,^30^ EoY is predominantly di-anionic at the pH of the diafiltrated samples (∼5–6). These negative charges promote strong electrostatic interactions with the cationic CTAB in the QS membrane, thereby enhancing encapsulation efficiency. Moreover, the E.E. for EoY in our QSs is much higher than that reported for fluorescein-incubated QSs in previous studies.^23^ In the case of fluorescein, removal of free dye during diafiltration also resulted in the complete loss of dye molecules attached to the nanovesicle surface. In our system, the presence of bromine atoms in EoY may reinforce its interaction with the lipid membrane, potentially enhancing its integration.^31^ In addition, the bromine substituents lower the p*K*_a_ of EoY compared to fluorescein, making EoY predominantly dianionic at the pH of QS formation (around 5), thereby further strengthening its electrostatic interaction with the positively charged membrane.^30^

Increasing the EoY concentration by more than twofold, from 140 µM to 320 µM, did not affect the encapsulation efficiency (Table S1, SI), which resulted high across both pre- and post-assembly loading approaches. This indicates that the QS maintain their loading capacity without signs of saturation within this concentration range.

### 2.1 Physicochemical characterization

**Fig. 3:**
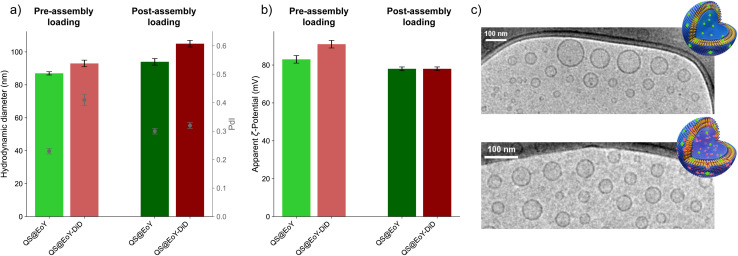
Physicochemical characterization of diafiltered QS dispersions using DLS, ELS, and cryo-TEM. (a) *Z*-average hydrodynamic diameter and PdI of the 4 QSs formulations. (b) Apparent *ζ*-potential of the 4 QSs formulations. (c) Representative cryo-TEM images of pre-assembly EoY-loaded QS dispersions, shown in the absence (top) and presence (bottom) of DiD in the membrane.

Dynamic Light Scattering (DLS) measurements were performed to determine the hydrodynamic diameter and the polydispersity index (PdI) of the QSs. Both preparation methods yielded nanoparticles with comparable *Z*-average values (80 to 100 nm) (Fig. 3b and S2, SI), indicating that the strategy of EoY incorporation, either during vesicle formation (pre-assembly loading) or by incubation (post-assembly loading), did not substantially alter the overall size. However, post-assembly dye-loaded QSs consistently showed slightly larger *Z*-average values than pre-assembly loaded ones, suggesting a modest increase in hydrodynamic diameter likely due to surface adsorption of the dye. PdI values were similar for the pre-assembly and post-assembly samples (≤0.41, Table S2, SI), reflecting a comparable size distribution. Only for QS@EoY we observed a larger PdI in the pre-assembly method suggesting a broader size distribution for this sample compared to that prepared following the post-assembly procedure. The *ζ*-potential of QSs was measured using Electrophoretic Light Scattering (ELS) to evaluate their colloidal stability and surface charge. All formulations exhibited highly positive *ζ*-potential values (Fig. 3b), consistent with the presence of cationic surfactants in the QS composition. The incorporation of EoY led to a slight decrease in *ζ*-potential when compared to blank CTAB-QS, likely due to electrostatic interactions between the anionic dye and the cationic QS surface. Both preparation methods yielded colloidally stable dispersions (*ζ*-potential >+25 mV). The pre-assembly loaded QSs displayed higher *ζ*-potential values than the post-assembly ones. This difference is consistent with a greater extent of EoY superficial adsorption in the post-assembly samples. Since the inner leaflet of the QS membrane is less accessible in the post-assembly loading method, a larger fraction of the anionic dye probably remained adsorbed on the outer surface of the nanovesicles, leading to a larger reduction in *ζ*-potential and also slightly higher hydrodynamic diameter. A summary of the DLS and ELS data is provided in Table S2, SI. Cryo-TEM measurements confirmed that QSs retained their expected unilamellar and spherical vesicular morphology, regardless of the presence of EoY and/or DiD. Representative micrographs in Fig. 3c show well-defined nanovesicles with a uniform size distribution and no significant aggregation, further supporting the colloidal stability of the prepared formulations.

### 2.2 Photophysical characterization

**Fig. 4:**
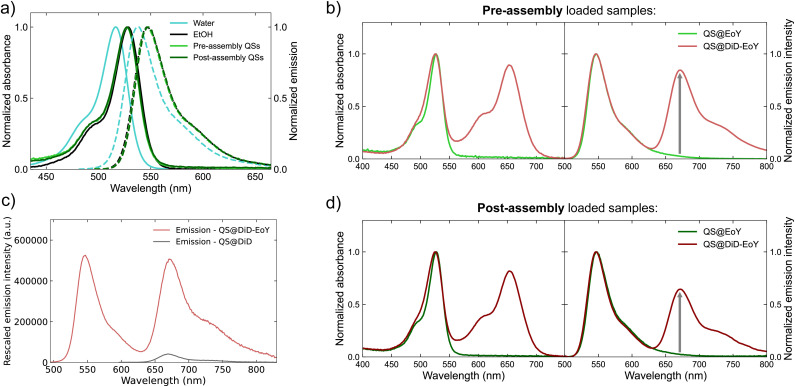
Spectroscopic characterization of QS dispersions (low concentration formulations). (a) Normalized UV-vis absorption (solid lines) and emission (dashed lines) spectra of EoY in different environments: distilled water, ethanol, and after loading into QS dispersions. (b) Normalized UV-vis absorption and emission spectra of EoY-loaded QS dispersions obtained *via* the pre-assembly loading method, both in the absence (green lines) and presence (red lines) of DiD in the membrane. (c) Absolute emission spectra of EoY-DiD-loaded QS dispersions and a reference dispersion of DiD-loaded QS, scaled by the absorbance of DiD at the excitation wavelength (475 nm). (d) Normalized UV-vis absorption and emission spectra of EoY-loaded QS dispersions obtained *via* the post-assembly dye loading method, both in the absence (green lines) and presence (red lines) of DiD in the membrane.

A comprehensive spectroscopic analysis was performed to investigate the interaction of EoY with QSs and compare the two preparation methods. Absorption and emission spectra were recorded for both pre-assembly loaded and post-assembly loaded EoY-QSs to assess the local environment of the dye. The absorption and emission spectra are identical for the two preparation methods (Fig. 4a) and closely resemble those of EoY in ethanol, showing a slight red shift compared to aqueous solution. This spectral behavior indicates that EoY experiences a less polar microenvironment than bulk water, consistent with preferential localization at the QS interface or within the membrane rather than in the aqueous lumen. While fluorescence quantum yield (*ϕ*) also varies between solvents (Table 1), it is influenced by multiple factors, including aggregation and quenching processes, and therefore spectral position provides a more reliable indicator of the local polarity experienced by the dye. Notably, the fluorescence quantum yield of EoY in QSs exceeds 30% for both preparation methods, higher than free EoY in water (25%). This high quantum yield, together with the preserved spectral bandshape, rules out significant dye aggregation within the nanovesicles at this dye concentration (138 µM). Additionally, the fluorescence lifetimes (*τ*) summarized in Table 1 (see the fluorescence decays in Fig. S3, SI), exhibit negligible differences between the two preparation methods, further confirming that EoY experiences a comparable microenvironment in both cases. Two-photon microscopy images acquired before and after diafiltration (Fig. S4, SI) show that fluorescence in both pre-assembly loaded and post-assembly loaded EoY samples is primarily localized within the dispersed nanostructures, with minimal background signal under identical acquisition conditions. These results indicate that EoY interacts strongly with the QSs during both preparation protocols and does not readily redistribute as free dye in solution.

**Table 1:** Spectroscopic properties of pre- and post-assembly dye-loaded QSs. Additional details on the lifetime decays fitting are reported in Table S4, SI

Sample	Donor quantum yield *ϕ*^a^ (%)		Donor fluorescence lifetime 〈*τ*〉^b^ (ns)	
EoY in water	25		1.13	
EoY in EtOH	67		2.90	
	Pre-assembly loading	Post-assembly loading	Pre-assembly loading	Post-assembly loading
QS@-EoY	32	33	1.71^c^	1.66^c^
QS@-DiD-EoY	2	5	1.01^d^	1.23^d^

a In presence of DiD, *ϕ* values were obtained by selectively exciting EoY at 490 nm and integrating only its band in the emission spectra.

b Fluorescence lifetimes were measured with excitation at 405 nm and emission collected at 550 nm.

c Average lifetimes 〈*τ*〉 calculated *via* a bi-exponential fit using the formula 
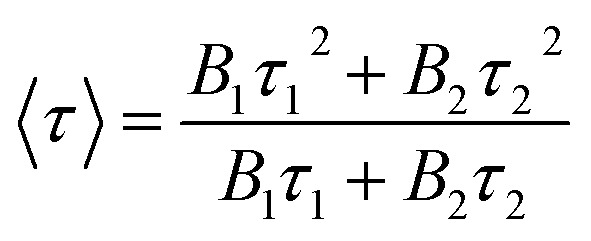
.

d Average lifetimes 〈*τ*〉 calculated *via* a tri-exponential fit using the formula 
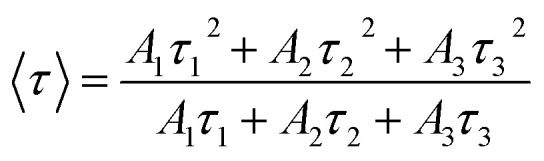
.

Concerning the QSs simultaneously loaded with EoY and DiD, the occurrence of FRET was confirmed by the detection of an intense DiD emission upon selective excitation of EoY at 475 nm. The relative contribution of the DiD emission band was more pronounced in pre-assembly loaded QSs (Fig. 4b) compared to post-assembly QSs (Fig. 4d). The direct excitation of DiD at 475 nm can be considered negligible, as shown in Fig. 4c, where a sample of DiD-loaded QSs (in the absence of EoY) exhibits very weak DiD emission if excited at the same excitation wavelength. The quantum yields and fluorescence lifetimes of the dispersions simultaneously loaded with EoY and DiD were reduced compared to the samples without the acceptor dye (see Table 1). This trend was observed for both preparation methods, providing further evidence of FRET in both systems. The presence of FRET offers additional proof of the successful encapsulation of EoY in both the pre-assembly loaded and post-assembly loaded QSs, as the process requires the energy donor and energy acceptor dyes to be within 1–10 nm of each other. The efficiency of the FRET process (*η*_FRET_) has been estimated from the fluorescence lifetime measurements, as shown in the following equation:
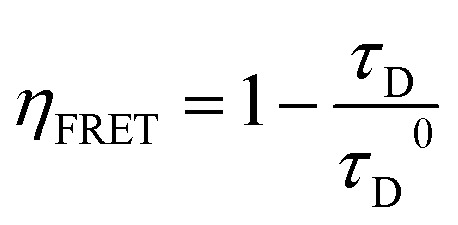
where *τ*_D_ is the donor fluorescence lifetime in the presence of the acceptor, while τ_D_^0^ is the donor fluorescence lifetime in the absence of the acceptor. The resulting *η*_FRET_ amount to 41% for pre-assembly loaded and 26% for post-assembly loaded QSs. The higher FRET efficiency in pre-assembly loaded QSs can be attributed to the presence of EoY in both the inner and outer leaflets of the bilayer, which increases the number of donor–acceptor interactions. In contrast, post-assembly loaded QSs should have EoY that primarily interacts with the external leaflet, thereby reducing the overall number of potential energy transfer events. Additionally, the slightly lower DiD concentration in post-assembly loaded QSs (66 µM *versus* 77 µM in pre-assembly loaded QSs, with similar EoY concentrations; see Table S1) may further contribute to the reduced FRET efficiency. The higher FRET efficiency observed in the dye-loaded sample represents also a proof that DiD is distributed in both leaflets of the membrane: in pre-assembly dye-loaded QSs, DiD molecules present in both leaflets of the membrane contribute to FRET signals, while in post-assembly dye loaded QSs, only DiD dyes present in the external leaflet are involved in FRET, and as consequence FRET is less efficient.

Fluorescence excitation spectra (Fig. S5, SI), recorded while selectively detecting DiD emission at 730 nm, further confirm the presence of FRET, as indicated by the distinct contribution of the EoY absorption band. Interestingly, the EoY band appears more pronounced in the excitation spectra compared to the corresponding absorption spectra. This effect is likely due to the formation of non-emissive DiD H-aggregates within the QSs membrane.^32,33^ While these aggregates contribute to the absorption spectrum, they do not appear in the excitation spectra because they cannot contribute to the detected emission of DiD.

At higher EoY concentrations (Fig. S5, SI), QSs loaded exclusively with EoY exhibit reduced fluorescence quantum yields and shorter lifetimes, consistent with dye aggregation within the nanovesicles (Table 1). In dispersions simultaneously loaded with EoY (at high concentration) and DiD, efficient FRET is maintained, as evidenced by characteristic acceptor emission upon donor excitation (bottom panels in Fig. S6, SI), indicating that FRET persists despite the energy donor self-association. The over time stability of EoY encapsulation was confirmed for at least one month after loading by absorption spectroscopy (Fig. S7, SI). The preserved absorption band shape indicates negligible EoY release, which would otherwise produce a blue-shifted spectrum.

## 3 Conclusions

We demonstrate, for the first time, the efficient incorporation of the hydrophilic dye Eosin Y into CTAB/cholesterol quatsomes, achieving high encapsulation efficiency (80–90%) and excellent retention without dye re-equilibration in water. Two different strategies to encapsulate EoY were employed: pre-assembly loading during vesicle production and incubation post vesicles production (post-assembly loading). In both cases, EoY loading does not alter the physicochemical properties of QSs, which preserve their size, morphology, and surface charge. Spectroscopic results indicate that EoY is located in a less polar microenvironment than bulk water, consistent with interfacial or membrane-associated localization, independently of the loading strategy. The resulting nanostructures exhibit favorable fluorescence properties, including high quantum yields at low dye concentration and efficient FRET in systems co-loaded with a hydrophobic membrane dye, supporting their applicability in bioimaging. Importantly, successful post-assembly loading highlights a flexible and scalable route for the on-demand functionalization of QSs, enabled by the strong affinity of EoY for the CTAB-based system. Overall, this work establishes QSs as versatile nanoplatforms for advanced optical applications, enabling the modular incorporation of hydrophilic and hydrophobic dyes and paving the way for the design of tailored fluorescent probes.

## 4 Technical section

Cetyltrimethylammonium bromide (CTAB, purity 99.0%) was purchased from Sigma-Aldrich (Saint Louis, USA). 5-Cholesten-3-ol (cholesterol, purity 95.0%) was purchased from PanReac AppliChem (Castellar del Vallès, Spain). 1,1′-Dioctadecyl-3,3,3′,3′-tetramethylindodicarbocyanine perchlorate (DiD) was bought from Life Technologies (Carlsbad, USA). Eosin Y (for microscopy) was obtained from Carlo Erba reagents S. A. S. All the purchased chemicals have been employed without further purification. All experiments were conducted using ultrapure Milli-Q water.

### 4.1 Preparation of quatsomes

The pre-assembly loaded dispersions were prepared using the DELOS-SUSP method (Depressurization of an Expanded Liquid Organic Solution Suspension), a patented compressed fluid technique that allows for the synthesis of nanovesicles in aqueous media from their membrane components.^18^ In a typical procedure, 79.88 mg of cholesterol was dissolved in 2.91 mL of HPLC-grade ethanol or 2.91 mL of a 970 µM DiD ethanolic solution, in the case of DiD-decorated samples. The vial was sealed with parafilm and heated in a water bath at 40 °C under stirring (750 rpm) for 20 minutes. Once the cholesterol was completely dissolved, the solution was transferred to a high-pressure vessel, with the vial washed using 0.2 mL of ethanol. The vessel was equipped with a heating mantle set to 35 °C, and after 10 minutes of heating, CO_2_ was introduced at a pressure of 115 bar. The system was left to equilibrate for one hour to ensure homogenization. Meanwhile, 72.48 mg of CTAB was dissolved in 25.10 mL of MilliQ water, or in an EoY solution in MilliQ water at the desired concentration (calculated to achieve a final volume of 28.22 mL). The CTAB aqueous solution was sealed with parafilm and heated in a bath at 40 °C under stirring (750 rpm) for 20 minutes. The high-pressure vessel was then slowly depressurized into the aqueous CTAB solution, while purging with dry nitrogen at 115 bar to maintain a constant pressure inside the reactor. The samples were stored at 4 °C and allowed to stabilize at least for 3 days. Following stabilization, the samples were purified by tangential flow filtration (TFF) and stored at 4 °C.

The post-assembly dye loaded QSs were prepared by adding hydrophilic dyes to blank or DiD-loaded QSs, which had been previously obtained using the DELOS-SUSP method. A suitable volume of EoY aqueous solution (ranging from 70 to 275 µL, depending on the stock solution concentration) was added to a dispersion of either blank or DiD-loaded QSs, achieving a total volume of 6 mL. The dispersions were incubated at room temperature and stirred at 750 rpm for 30 minutes, after which 5 mL of each incubated sample was diafiltrated and stored at 4 °C.

All purified samples in the context of this study were processed using C04-E100-05 N hollow fiber columns (Repligen) with a 100 kDa molecular weight cut-off, operated at a transmembrane pressure of 5 psi and a flow rate of 27 mL min^−1^. Diafiltration was performed using a KrosFlo® Research IIi tangential flow filtration (TFF) system.

### 4.2 Spectroscopic measurements

All solutions for spectroscopic measurements were prepared using spectrophotometric or HPLC-grade solvents. UV-vis absorption spectra were recorded with a PerkinElmer Lambda 650 spectrophotometer, while steady-state fluorescence excitation and emission spectra were acquired using an FLS1000 fluorometer (Edinburgh Instruments). Fluorescence measurements were performed on diluted samples (absorbance <0.1 at the absorbance maximum) and corrected for both the wavelength-dependent excitation intensity and detector response. For highly scattering samples, suitable long-pass filters were inserted in the emission path to remove interference from scattered excitation light. Fluorescence quantum yields were determined using 1 cm pathlength quartz cuvettes, with fluorescein in 0.1 M NaOH aqueous solution (quantum yield *Φ* = 0.90) as the reference standard. Time-resolved fluorescence decays were acquired by time-correlated single photon counting (TCSPC), exciting the samples with a 405 nm pulsed diode laser (pulse width ∼200 ps, repetition rate 1 MHz). The time-resolved fluorescence decays were employed to estimate *η*_FRET_, as described in the discussion. In principle *η*_FRET_ can be estimated also from the fluorescence quantum yields and from the comparison between excitation and absorption spectra, but the fluorescence lifetime measurements are generally considered as the most reliable method for FRET analysis, as they directly probe the donor's excited-state decay, which is intrinsically affected by energy transfer.^21,34^ Indeed, the other methods to determine *η*_FRET_ can be influenced by additional quenching processes (such as static quenching) that do not alter the donor excited-state decay kinetics. Moreover, variations in local concentration do not significantly affect the FRET efficiency calculated using the method based on fluorescence lifetimes. While detecting small lifetime variations can be challenging when donor lifetimes are very short, this limitation does not apply to EoY in these conditions, whose lifetimes exceed 1 ns. This sufficiently long decay time allows for the resolution of FRET-induced changes using our experimental setup. Overall, lifetime-based methods provide a more direct and accurate assessment of FRET efficiency, *i.e.* reducing artifacts from aggregation phenomena.

#### 4.2.1 Encapsulation efficiency estimation

The actual concentration of dyes in the QSs dispersions was determined using the Lambert–Beer law, following complete disruption of the vesicles by dilution in ethanol (1 : 50 v/v). This treatment ensured full solubilization of all components. It was assumed that the molar extinction coefficients of the dyes were unaffected by the presence of solubilized QSs components and matched their values in pure ethanol. The extinction coefficients used were *ε* = 246 000 M^−1^ cm^−1^ at 648 nm for DiD^19^ and *ε* = 88 000 M^−1^ cm^−1^ at 525 nm for EoY (determined from the Beer–Lambert law using serial dilutions of EoY stock solutions of known concentration). The dye concentrations obtained from UV-vis absorption measurements were then used to calculate the encapsulation efficiency (E.E.), defined as the fraction of dye retained in the nanoparticle dispersion after the diafiltration process:1
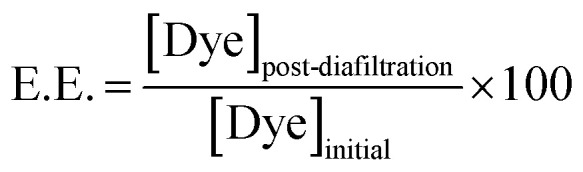
where [Dye]_initial_ and [Dye]_post-diafiltration_ correspond to the concentrations before and after the diafiltration process performed after either pre- or post-assembly loading. This purification step is expected to remove all free dye and surfactant molecules in solution, as schematically depicted in Fig. 2, and may also partially eliminate dyes electrostatically adsorbed on the outer surface of the nanovesicles.

### 4.3 Two-photon microscopy

Two-photon fluorescence imaging of EoY-loaded QS dispersions was performed using a Nikon A1R MP+ upright two-photon microscope equipped with a Coherent Chameleon Discovery femtosecond pulsed laser (pulse duration 100 fs, repetition rate 80 MHz, tunable excitation range 700–1300 nm). A 25× water-dipping objective (numerical aperture 1.1, working distance 2 mm) was used for both excitation and fluorescence collection. The emitted fluorescence was detected in non-descanned mode using a high-sensitivity GaAsP detector, preceded by a filter cube selecting the emission window between 506 and 593 nm. QSs dispersions were analyzed in quartz cuvettes placed horizontally under the microscope objective, exciting the sample at 1000 nm. Each cuvette was completely filled with the sample to eliminate air gaps between the upper cuvette wall and the liquid surface. Distilled water was used to maintain optical contact between the objective and the cuvette surface.

### 4.4 Cryo-TEM

Specimens were rapidly vitrified by plunge-freezing in liquid ethane at 94 K, ensuring preservation of their native structure within a thin layer of vitreous ice. Cryo-TEM micrographs were acquired at the Servei de Microscòpia of the Universitat Autònoma de Barcelona using the JEOL JEM-2011 microscope operating at 200 kV under low-dose conditions.

### 4.5 Dynamic and electrophoretic light scattering

Dynamic Light Scattering (DLS) measurements were performed at 25 °C on undiluted samples using 1 mL polystyrene cuvettes and a Zetasizer Ultra instrument (Malvern), equipped with a 633 nm He–Ne laser and a fluorescence filter to enable measurements on samples containing fluorescent dyes absorbing at the laser wavelength. Data were acquired in backscattering mode, with the detector set at an angle of 173°.

Electrophoretic Light Scattering (ELS) was used to determine the electrophoretic mobility of the samples, using the same Zetasizer Ultra equipment. Measurements were carried out at 25 °C, with scattered light collected in forward detection mode (13°). The *ζ*-potential (*ζ*) was estimated from the measured electrophoretic mobility using the Helmholtz—Smoluchowski approximation, and thus the reported values correspond to apparent *ζ*-potentials. For each sample, three independent measurements were performed.

## Author contributions

Conceptualization: CS, MK, AD, GVN. Methodology: MK, AD, GVN. Formal analysis: AD, GVN, GC. Investigation: AD, GVN, GC. Data curation: AD, GVN, GC. Writing – original draft: AD, GVN, CS. Writing – review & editing: all authors. Funding acquisition: CS, MK, NV. Supervision: NV, CS, MK. Project administration: NV, CS, MK.

## Conflicts of interest

There are no conflicts to declare.

## Supplementary Material

NA-008-D6NA00124F-s001

## Data Availability

All data discussed in this manuscript are included in the main text and in the supplementary information (SI). Supplementary information: sample compositions, additional dynamic light scattering and spectroscopic data, and two-photon microscopy measurements. See DOI: https://doi.org/10.1039/d6na00124f.
