# Supplementary material for: Quatsomes as versatile fluorescent nanocarriers: stable Eosin Y loading and FRET with a membrane dye
Source: Nanoscale Adv. 2026 May 15;8(13):3693–701. doi: 10.1039/d6na00124f (PMC13195686; doi:10.1039/d6na00124f)
Supplement: NA-008-D6NA00124F-s001 [file NA-008-D6NA00124F-s001.pdf]

Supplementary Information (SI) for Nanoscale Advances.
This journal is © The Royal Society of Chemistry 2026

Supporting Information for Quatsomes as Versatile Fluorescent Nanocarriers: Stable Eosin Y Loading and FRET with a Membrane Dye

Andrea Delledonne,[a‡] Guillem Vargas-Nadal,[b,c‡], Giacomo Cotelli,[a] Nora Ventosa,[b,c] Mariana Köber,[b,c*] and Cristina Sissa[a*]

[a] Dipartimento di Scienze Chimiche, della Vita e della Sostenibilità Ambientale, Università di Parma, Parco Area delle Scienze 17A, 43124, Parma, Italy, [b] Institute of Materials Science of Barcelona (ICMAB-CSIC), Universitat Autònoma de Barcelona, 08193 Barcelona, Spain; [c]Centro de Investigación Biomédica en Red in the Subject Area of Bioengineering, Biomaterials and Nanomedicine (CIBER-BBN), 28029 Madrid, Spain

S1 Materials and Methods

Table S1: Summary of the initial concentrations of the components used to prepare the QS suspensions. The concentrations of the dyes after diafiltration and the corresponding encapsulation efficiencies are also reported.

		**Initial concentrations**				**Encapsulated dyes**		**E.E.**	
**Preparation method**	**Sample**	**Cholesterol**	**CTAB**	**EoY**	**DiD**	**EoY**	**DiD**	**EoY**	**DiD**
Pre-assembly	QS@EoY	7 mM	7 mM	138 μM	-	113 μM	-	82%	-
	QS@EoY-DiD	7 mM	7 mM	138 μM	100 μM	129 μM	77 μM	94%	77%
	QS@EoY high conc.	7 mM	7 mM	320 μM	-	281 μM	-	88%	-
	QS@EoY-DiD high conc.	7 mM	7 mM	320 μM	100 μM	308 μM	77 μM	96%	77%
Post-assembly	QS@EoY	7 mM	7 mM	138 μM	-	103 μM	-	75%	-
	QS@EoY-DiD	7 mM	7 mM	138 μM	100 μM	123 μM	66 μM	89%	66%
	QS@EoY high conc.	7 mM	7 mM	320 μM	-	290 μM	-	91%	-
	QS@EoY-DiD high conc.	7 mM	7 mM	320 μM	100 μM	298 μM	60 μM	93%	60%

S2 Physicochemical characterization

Table S2: DLS and ELS results of QS suspensions as the average of three measurements with the corresponding standard error of the mean (SEM). Measurements were performed at 25°C as described in the technical section of the main text.

Sample Type	Sample	Hydrodynamic diameter[a] (nm)	PdI	Apparent ζ-potential[b] (mV)
-	Plain QS	91 ± 1	0.4 ± 0.02	94 ± 1
Pre.assembly	QS@EoY	87 ± 1	0.23 ± 0.01	83 ± 2
	QS@EoY-DiD	93 ± 2	0.41 ± 0.02	91 ± 2
	QS@EoY high conc.	88 ± 2	0.30 ± 0.01	87 ± 2
	QS@EoY-DiD high conc.	97 ± 2	0.37 ± 0.01	97 ± 1
Post-assembly	QS@EoY	94 ± 2	0.30 ± 0.01	78 ± 1
	QS@EoY-DiD	105 ± 2	0.32 ± 0.01	78 ± 1
	QS@EoY high conc.	90 ± 1	0.31 ± 0.01	84 ± 2
	QS@EoY-DiD high conc.	111 ± 2	0.33 ± 0.02	77 ± 2

[a] *Obtained from the corresponding peak in the particle size distribution by intensity.*
[b] *Apparent ζ-potential calculated using the Helmholtz–Smoluchowski equation.*

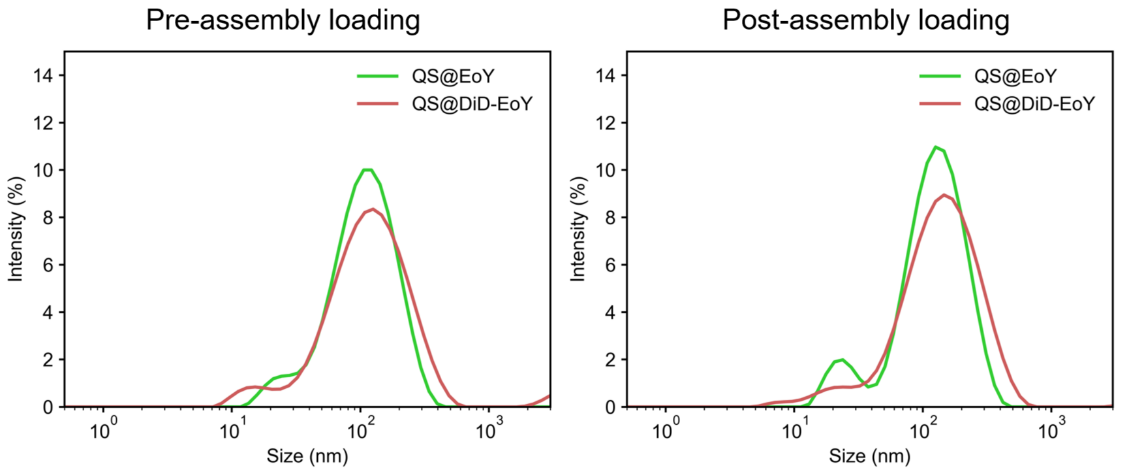

Figure S1: DLS intensity-weighted size distributions of the lower concentration samples reported in Table S2.

S3 Spectroscopic Data

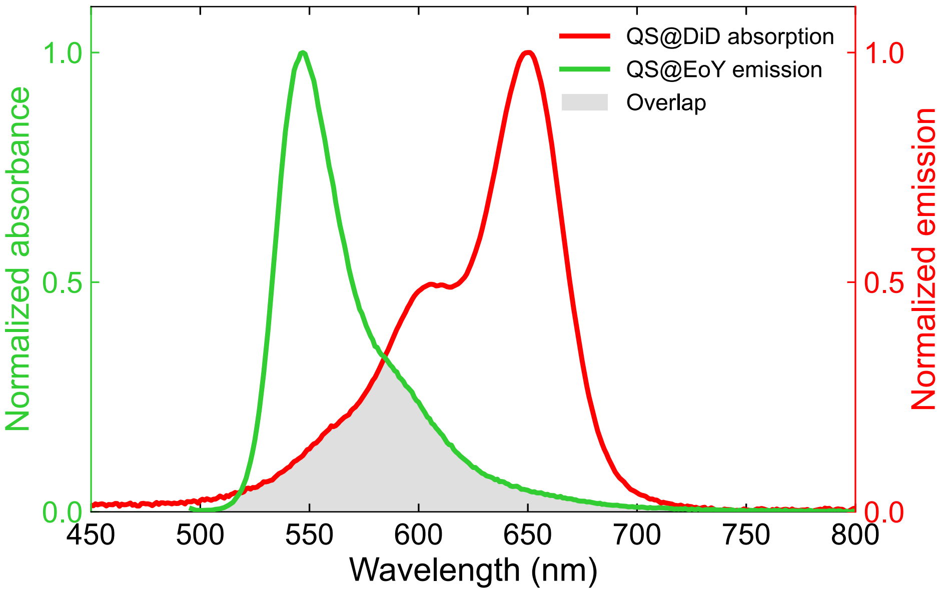

Figure S2: Comparison between the absorption spectrum of QSs loaded only with DiD (dye concentration 100 μM) and the emission of QSs loaded only with EoY (dye concentration 100 μM).

Table S3: Spectroscopic properties of pre-assembly and post-assembly QSs with higher concentration of EoY (320 μM).

	Donor quantum yield ϕ^a (%)		Donor fluorescence lifetime $\langle\tau\rangle^b$ (ns)	
Sample	Pre-assembly	Post-assembly	Pre-assembly	Post-assembly
QS@-EoY (high conc.)	9	8	0.62	0.57
QS@-DiD-EoY (high conc.)	2	3	0.62	0.45

[a] *In presence of DiD, ϕ values were obtained by selectively exciting EoY at 490 nm and integrating only its band in the emission spectra.*
[b] *Fluorescence lifetimes were measured with excitation at 405 nm and emission collected at 550 nm. Average lifetimes $\langle\tau\rangle$ calculated via a tri-exponential fit using the formula* $\langle\tau\rangle = \frac{A_1\tau_1^2+A_2\tau_2^2+A_3\tau_3^2}{A_1\tau_1+A_2\tau_2+A_3\tau_3}$.

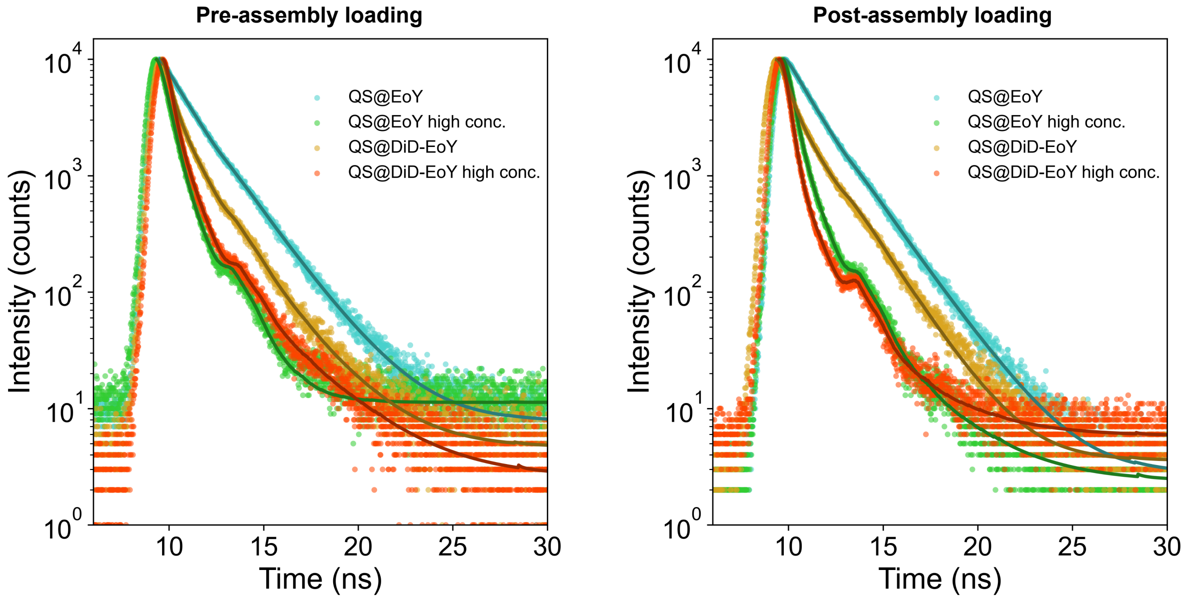

Figure S3: Fluorescence lifetime decays of Eosin Y in Quatsomes prepared via pre-assembly (left) and post-assembly (right) loading. Measurements were performed using a 405 nm picosecond-pulsed laser for excitation, and emission was collected at 550 nm, corresponding to the EoY fluorescence maximum. The darker lines correspond to the fitting lines obtained through reconvolution exponential components fitting, whose corresponding optimized parameters are reported in Table S4.

Table S4: Optimized parameters obtained using a reconvolution exponential components fitting from the FAST software

Sample	A_1 (ns)	τ_1 (ns)	A_2 (ns)	τ_2 (ns)	A_3 (ns)	τ_3 (ns)	$\langle\tau\rangle$ (ns)	χ^2
QS@-EoY pre-assembly	0.0146 (44%)	1.185	0.0105 (56%)	2.114	-	-	1.71	1.311
QS@-EoY post-assembly	0.0128 (38%)	1.098	0.0116 (62%)	2.006	-	-	1.66	0.620
QS@DiD-EoY pre-assembly	0.0336 (38%)	0.283	0.0099 (45%)	1.116	0.0018 (17%)	2.366	1.01	1.447
QS@DiD-EoY post-assembly	0.0252 (42%)	0.449	0.0090 (58%)	1.778	-	-	1.23	1.047
QS@-EoY (high conc.) pre-assembly	0.0332 (48%)	0.278	0.0123 (48%)	0.761	0.0002 (4%)	2.763	0.62	1.187
QS@-EoY (high conc.) post-assembly	0.0411 (63%)	0.292	0.0069 (33%)	0.901	0.0002 (4%)	2.907	0.57	0.455
QS@-DiD-EoY (high conc.) pre-assembly	0.0545 (52%)	0.179	0.0101 (40%)	0.755	0.0005 (8%)	2.873	0.62	1.267
QS@-DiD-EoY (high conc.) post-assembly	0.0592 (77%)	0.218	0.0036 (19%)	0.915	0.0002 (4%)	3.001	0.45	1.426

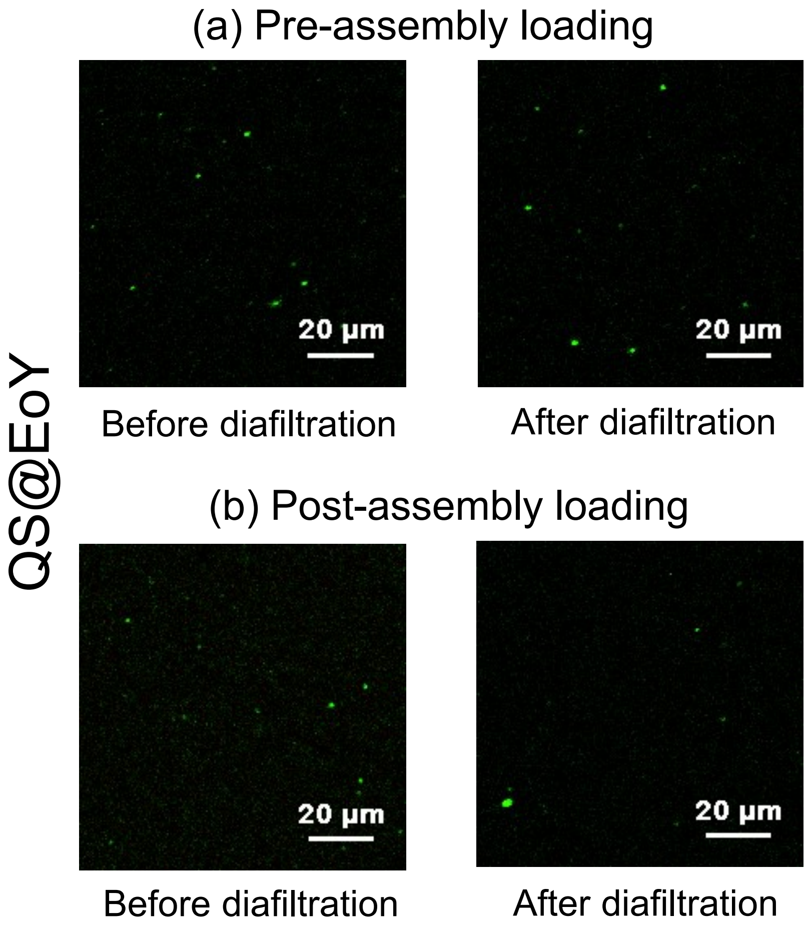

Figure S4: Two-photon microscopy images of EoY-labelled Quatsomes prepared via pre-assembly (panel a) and post-assembly (panel b) loading, shown both before and after diafiltration. All images were acquired under identical conditions using 1000 nm excitation at 3% laser power.

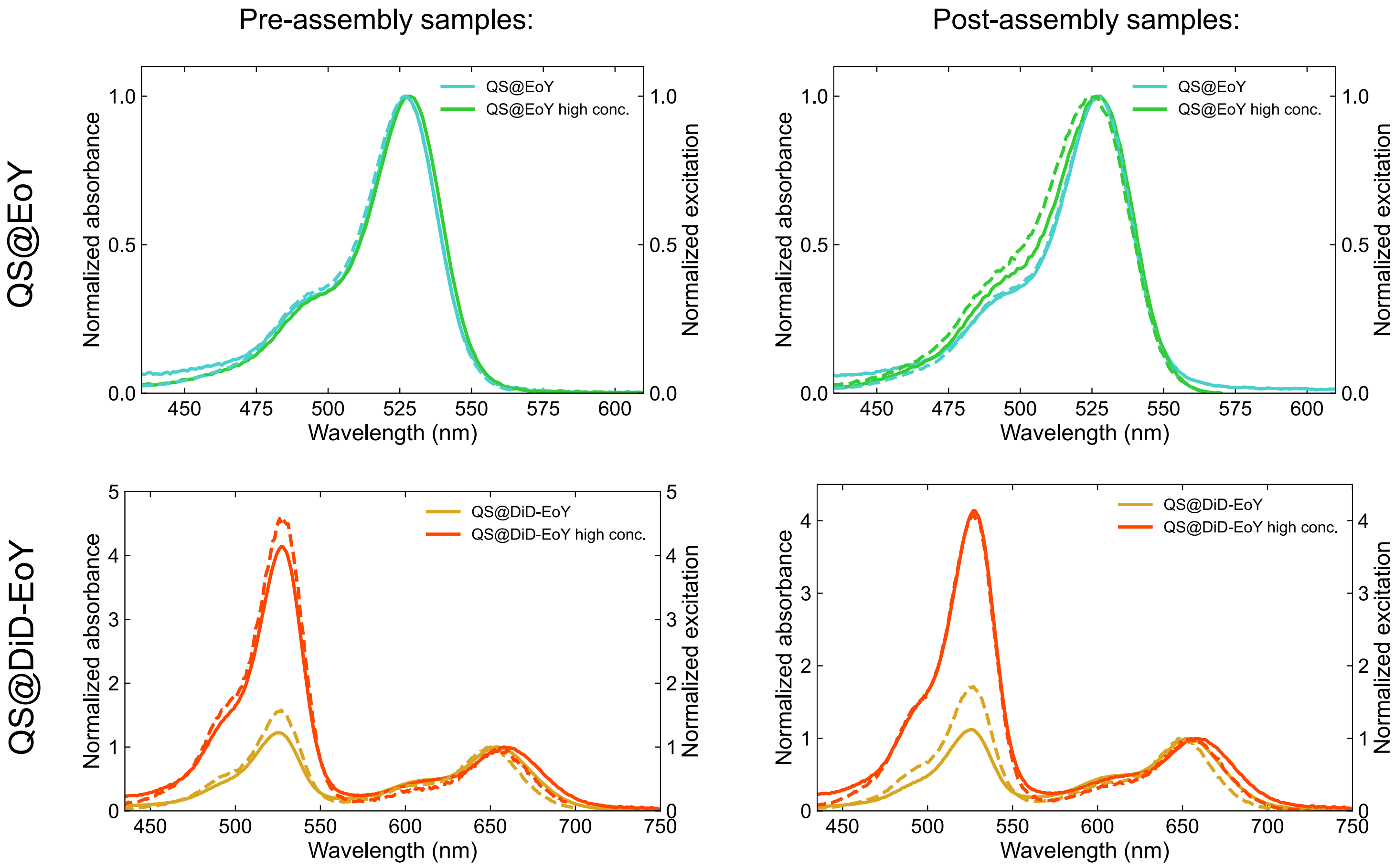

Figure S5: Comparison of absorbance (solid lines) and excitation spectra (dashed lines) for pre-assembly (left) and post-assembly (right) Quatsome formulations. Excitation spectra were recorded by monitoring EoY emission at 575 or 590 nm (for QS@EoY), and DiD emission at 700 or 730 nm (for QS@DiD-EoY samples).

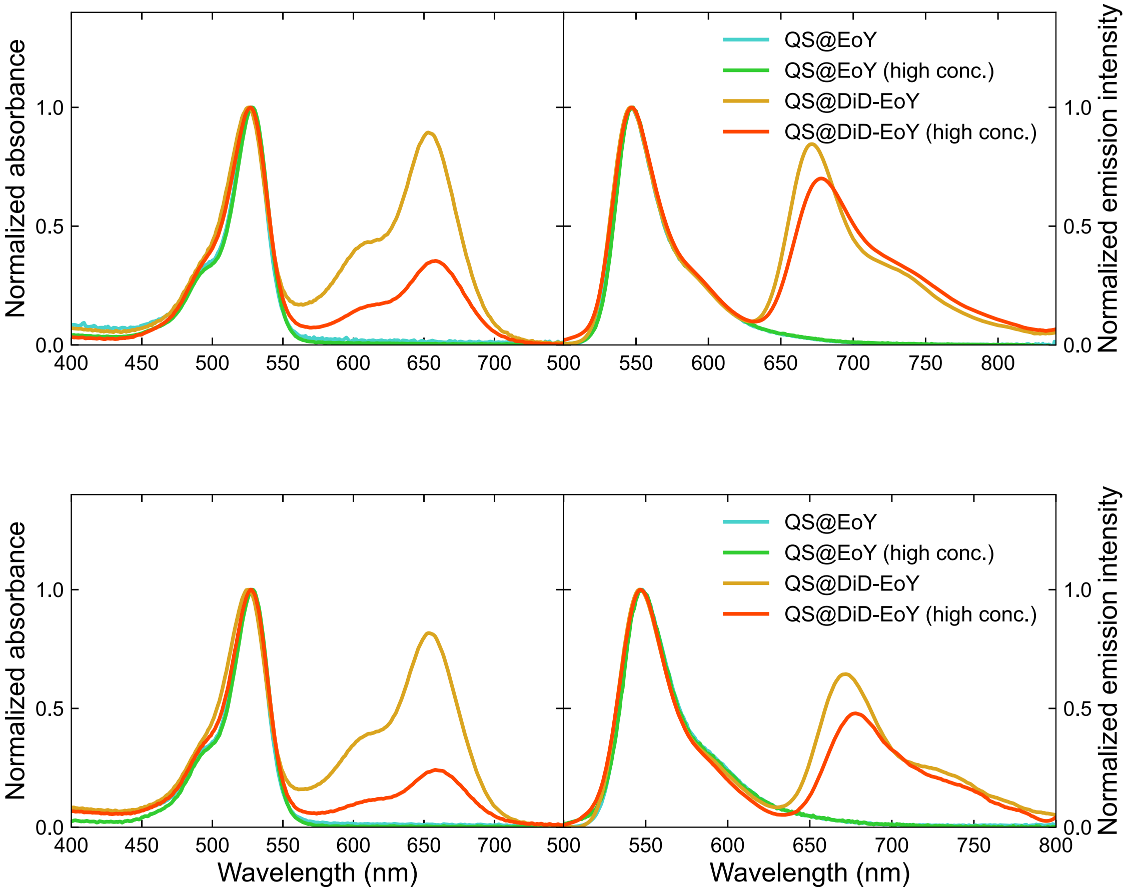

Figure S6: Absorption and emission spectra of pre-assembly (left) and post-assembly (right) QSs samples, prepared with two different concentrations of the energy donor dye Eosin Y (EoY), with and without the presence of the energy acceptor dye DiD. Emission spectra were recorded upon selective excitation of EoY: at 490 nm for EoY-only samples and at 475 nm for samples containing both EoY and DiD. All emission spectra are normalized to the energy donor (EoY) emission intensity to facilitate the qualitative comparison of FRET efficiency.

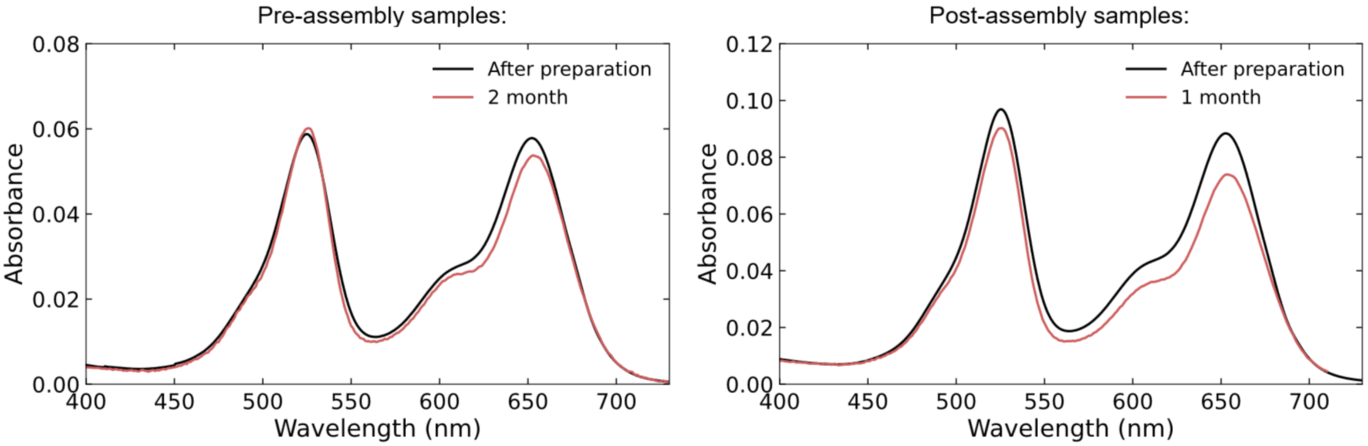

Figure S7: Time-dependent stability of QSs prepared with EoY and DiD, assessed by comparing absorption spectra recorded after preparation and after 1 or 2 months. Pre-assembly and post-assembly samples were diluted 1:200 and 1:120, respectively, before measurement.
